# Comprehensive linker-scanning mutagenesis of the hepatitis C virus E1 and E2 envelope glycoproteins reveals new structure–function relationships

**DOI:** 10.1099/vir.0.034314-0

**Published:** 2011-10

**Authors:** Małgorzata Rychłowska, Ania M. Owsianka, Steven K. H. Foung, Jean Dubuisson, Krystyna Bieńkowska-Szewczyk, Arvind H. Patel

**Affiliations:** 1 Department of Molecular Virology, University of Gdańsk, Kładki 24, 80-822 Gdańsk, Poland; 2 MRC–University of Glasgow Centre for Virus Research, 8 Church Street, Glasgow G11 5JR, UK; 3 Department of Pathology, Stanford University School of Medicine, Stanford, CA 94304, USA; 4 Institut Pasteur de Lille, Centre for Infection & Immunity of Lille (CIIL), F-59019 Lille, France; 5 Inserm U1019, F-59019 Lille, France; 6 CNRS UMR8204, F-59021 Lille, France; 7 Université Lille Nord de France, F-59000 Lille, France

## Abstract

Despite extensive research, many details about the structure and functions of hepatitis C virus (HCV) glycoproteins E1 and E2 are not fully understood, and their crystal structure remains to be determined. We applied linker-scanning mutagenesis to generate a panel of 34 mutants, each containing an insertion of 5 aa at a random position within the E1E2 sequence. The mutated glycoproteins were analysed by using a range of assays to identify regions critical for maintaining protein conformation, E1E2 complex assembly, CD81 receptor binding, membrane fusion and infectivity. The results, while supporting previously published data, provide several interesting new findings. Firstly, insertion at amino acid 587 or 596 reduced E1E2 heterodimerization without affecting reactivity with some conformation-sensitive mAbs or with CD81, thus implicating these residues in glycoprotein assembly. Secondly, insertions within a conserved region of E2, between amino acid residues 611 and 631, severely disrupted protein conformation and abrogated binding of all conformation-sensitive antibodies, suggesting that the structural integrity of this region is critical for the correct folding of E2. Thirdly, an insertion at Leu-682 specifically affected membrane fusion, providing direct evidence that the membrane-proximal ‘stem’ of E2 is involved in the fusion mechanism. Overall, our results show that the HCV glycoproteins generally do not tolerate insertions and that there are a very limited number of sites that can be changed without dramatic loss of function. Nevertheless, we identified two E2 insertion mutants, at amino acid residues 408 and 577, that were infectious in the murine leukemia virus-based HCV pseudoparticle system.

## Introduction

Hepatitis C virus (HCV) is an enveloped, positive-sense RNA virus belonging to the genus *Hepacivirus* in the family *Flaviviridae*. The envelope glycoproteins E1 and E2 are present on the surface of the virus particle and are major players during infection of host cells. Both are type 1 transmembrane proteins containing a highly glycosylated N-terminal ectodomain and a C-terminal transmembrane domain that anchors them to the membranes of the endoplasmic reticulum (ER) (Brass *et al.*, 2006; Helle & Dubuisson, 2008). E1 and E2 assemble in the ER as a non-covalent heterodimer, which was thought to be the functional complex responsible for host cell attachment and entry (Bartosch *et al.*, 2003b; Dubuisson, 2007), but it has recently been shown that glycoproteins in the envelope of infectious virions form large covalent complexes that are stabilized by disulphide bridges (Vieyres *et al.*, 2010). Entry involves multiple interactions of viral particles with a set of co-receptors required at sequential entry steps of attachment, binding, endocytosis and fusion (Benedicto *et al.*, 2008; Helle & Dubuisson, 2008; Ploss *et al.*, 2009). The tetraspanin CD81, scavenger receptor class B type I, and the tight junction proteins claudin-1 and occludin have all been identified as being essential for virus entry, but direct binding of the E1E2 heterodimer has so far been confirmed only for CD81 (Cocquerel *et al.*, 2003). Owing to the association of HCV particles with very low density lipoproteins during virus assembly and secretion, lipoprotein receptors are also important entry factors (Burlone & Budkowska, 2009). Various structural and functional features of the E1E2 heterodimer have been described, including residues involved in heterodimerization (Ciczora *et al.*, 2005; Cocquerel *et al.*, 1998, 2000; Drummer & Poumbourios, 2004; Michalak *et al.*, 1997; Op De Beeck *et al.*, 2000; Patel *et al.*, 2001; Selby *et al.*, 1994), CD81-receptor binding (Drummer *et al.*, 2006; Owsianka *et al.*, 2006; Rothwangl *et al.*, 2008) and the ER retention signal (Cocquerel *et al.*, 1998). Fusion mediated by HCV glycoproteins is pH dependent and probably occurs after endocytosis in early endosomes (Blanchard *et al.*, 2006; Meertens *et al.*, 2006; Tscherne *et al.*, 2006). The similarity of the genomic organization of HCV to other members of the family *Flaviviridae* suggests that E2 is a class II fusion protein, with a candidate fusion loop at amino acid residues 502–520 (Krey *et al.*, 2010). However, several other hydrophobic domains within E1 and E2 have been investigated as potential fusion peptides (Lavillette *et al.*, 2007; Perez-Berná *et al.*, 2006; Russell *et al.*, 2009; Spadaccini *et al.*, 2010). It is still unclear which of the two partners is the fusion protein, and how the heterodimer mediates membrane fusion.

To fulfil such a multitude of biological functions, E1 and E2 must contain different segments with distinct properties. In the absence of a high-resolution structure, mutational analysis can provide valuable insights into the relationship between structure and function. In this study we constructed a panel of mutants with insertions randomly distributed throughout E1 and E2, and subjected them to a comprehensive functional analysis by using a hierarchy of assays – from protein folding to infectivity – in order to identify and delineate functional domains.

## Results and Discussion

### Construction and expression of E1 and E2 insertion mutants

**Fig. 1.**
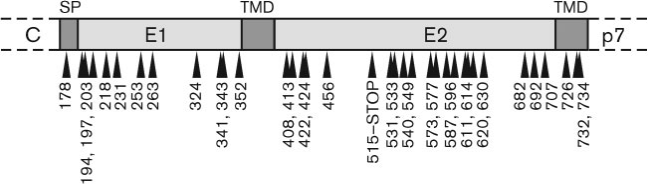
Schematic representation of insertions in HCV E1 and E2 proteins. Arrows indicate the position of each 5 aa insertion. Numbers correspond to the amino acid immediately preceding the insertion in the HCV polyprotein. The insertion at amino acid position 515 encodes a stop codon, resulting in a truncated E2 protein. Also shown are the E1 signal peptide (SP), and the transmembrane domains (TMD).

Linker-scanning mutagenesis was employed to generate a library of 5 aa insertions in E1 and E2. Briefly, an *in vitro* transposition reaction was used to introduce a 15 bp insertion at random into genotype 1a strain H77c E1E2 cDNA, resulting in a single 5 aa insertion in the protein. Fifty insertion mutants were isolated, of which 35 encoded ‘read-through’ mutations while 15 contained premature stop codons. The read-through insertions were distributed fairly evenly, with 11 located in E1, one in the E1 signal peptide and 23 in E2 (Fig. 1). The identity of the amino acids encoded by the insertions is given in Table 1. Mutants were numbered according to the amino acid position of the viral polyprotein immediately N-terminal to the insertion site. Natural glycosylation sites were preserved in all mutants except *in*577, which lacked the glycosylation site at Asn-576 (Table 1). We selected the 34 read-through mutants and compared their properties with those of wild-type (WT) E1E2 across a range of functions, from the fundamental characteristics of correct folding and heterodimerization to the higher level functions of fusion and infectivity. The results of all these experiments are summarized and correlated in Table 2. As an extra positive control, in addition to WT, we included *in*178, which had an insert in the E1 signal peptide and therefore expressed WT E1E2 glycoprotein. All experiments included a negative control that lacked E1 and E2 glycoproteins, and, in addition to this no-glycoprotein control, as a second E2-negative control we used the mutant *in*515-STOP, which has a premature stop codon at amino acid 515 and therefore expresses a truncated E2 protein.

**Table 1.** Amino acid sequence of insertions Inserted amino acids are shown in italics. All *N*-linked glycosylation sites are in boldface type.

Mutant*	Insertion context
*in*178	SFSIFL *FKHFL* LALL
*in*194	SAYQV *FKHQV* R**N**SS
*in*197†	QVR**N**S *CLNNS*SGLY
*in*203	GLYHV *FKHHV* TNDC
*in*218	EAAD *VFKHD* AILHT
*in*231	PCVR *CLNIR* EG**N**AS
*in*253	RDGKL *FKHK* LPTTQ
*in*263	RRHID *CLNID* LLVGS
*in*324	DMMM *FKQMM* NWSP
*in*341	LLRIP *LFKHP* QAIMD
*in*343	RIPQA *CLNKA* IMDMI
*in*352	IAGAH *CLNTH* WGVL
*in*408	PGAK *LFKHK* QNIQL
*in*413	QNIQL *MFKQL* INT**N**GS
*in*422	GSWHI *MFKHI***N**STAL
*in*424†	WHI**N**S *CLNNS*TALNC
*in*456	CPERL *VFKQL* ASCRR
*in*515	TPSPVV STOP
*in*531	SWGA *MFKHA***N**DTD
*in*533†	GA**N**D *CLNND*TDVFV
*in*540†	FVL**N***CLNIN*NTRPP
*in*549	GNW *CLNNW* FGCT
*in*573	VIGG *CLNRG* VG**N**N
*in*577‡	VG**N**N *MFKHN* TLLCP
*in*587	DCFR *CLNIR* KHPEA
*in*596	TYSR *CLNTR* CGSG
*in*611	MVDY *LFKHY* PYRL
*in*614	YPYR *LFKHR* LWHY
*in*620	HYPC *CLNTC* TI**N**YT
*in*630	IFKVR *CLNIR* MYVGG
*in*682	SFTTL *LFKHL* PALS
*in*692	LIHL *LFKHL* HQNIV
*in*707	GVGS *MFKQS* SIASW
*in*726	LFLLL *FKHLL* ADARV
*in*732	ARVC *CLNIC*S CLWM
*in*734	VCSC *CLNTC* LWMM

* Mutants are numbered according to the amino acid position immediately N-terminal of the insertion site.

† For mutants *in*197 *in*424, *in*533 and *in*540 the glycosylation motif is reconstituted at the end of the insertion, as underlined.

‡ Mutant *in*577 has lost glycosylation site Asn-576.

**Table 2.** Summary of mutant E1E2 protein characteristics

Mutant	E1E2 expression*		E2 folding (conformation-sensitive mAb binding*)					E1E2 hetero-dimerization†	CD81 binding*	Fusion‡	HCVpp incorporation†	HCVpp infectivity§
	H111 AP21.010 binding	AP33 ALP98 binding	H35/H48	H53	CBH-4B/4D/4G	CBH-5	CBH-7					
*in*178	+++	+++	+++	+++	+++	+++	+++	+++	+++	85 %	+++	70 %
*in*194	++	+++	+++	+++	+++	+++	+++	?	+++	61 %	?	7 %
*in*197	+++	+++	+++	+++	+++	+++	+++	?	+++	7 %	?	−
*in*203	++	+++	+++	+++	+++	+++	+++	?	+++	−	?	−
*in*218	+++	+++	+++	+++	+++	+++	+++	+++	+++	−	+	−
*in*231	+++	+++	+++	+++	+++	++	+++	+++	+++	−	+++	−
*in*253	+++	+++	+++	+++	+++	+++	+++	+++	+++	17 %	+++	3 %
*in*263	+++	+++	+++	+++	+++	+++	+++	+++	+++	−	+	−
*in*324	+++	+++	+++	+++	+++	+++	+++	+++	+++	−	−	−
*in*341	+++	+++	+++	+++	+++	++	+++	+++	+++	−	+++	−
*in*343	+++	+++	+++	+++	+++	++	+++	+++	++	42 %	++	−
*in*352	++	+++	+++	+++	+++	+++	+++	+++	+++	35 %	+++	−
*in*408	+++	+++	+++	+++	+++	+++	+++	+++	+++	159 %	+++	20 %
*in*413	+++	+++	+++	+++	+++	+++	+++	+++	+++	7 %	++	−
*in*422	+++	+++	+++	+++	+++	++	+++	+++	+	11 %	+++	−
*in*424	+++	+++	+++	+++	+++	+++	+++	+++	+	−	+++	−
*in*456	+++	+++	+++	+++	+++	+++	+++	+++	+++	9 %	−	−
*in*515 STOP	+++	+	−	−	−	−	−	−	−	−	−	−
*in*531	+++	+++	+	+++	++	−	+++	+++	+	−	+++	−
*in*533	+++	+++	+	+++	+++	−	+++	+++	+	−	+++	−
*in*540	+++	+++	++	+	+	−	−	+	++	−	−	−
*in*549	+++	+++	++	−	−	−	−	+	++	−	−	−
*in*573	+++	+++	+++	+++	+++	+++	+++	+++	+++	7 %	++	−
*in*577	+++	+++	+++	+++	+++	+++	+++	++	+++	130 %	+++	13 %
*in*587	+++	+++	+++	+++	−	+	++	+	+++	−	−	−
*in*596	+++	+++	+++	+++	+	+++	++	+	++	−	−	−
*in*611	+++	+++	−	−	−	−	−	+++	+	−	−	−
*in*614	+++	+++	+	+	−	−	−	+++	+	−	+++	−
*in*620	+++	+++	−	+	−	−	−	+++	+	−	−	−
*in*630	+++	+++	+	−	−	−	−	+++	+	−	−	−
*in*682	+++	+++	+++	+++	+++	+++	+++	+++	+++	−	+++	−
*in*692	+++	+++	+++	+++	+++	+++	+++	−	+++	−	−	−
*in*707	+++	+++	+++	+++	+++	+++	+++	−	+++	−	−	−
*in*726	+++	+++	+++	+++	+++	+++	+++	−	++	−	++	−
*in*732	+++	+++	+++	+++	+++	+++	+++	+++	+++	−	−	−
*in*734	+++	+++	+++	+++	+++	+++	+++	+++	+++	−	−	−

* Relative to WT: −, <15 %; +, 15–50 %; ++, 51–75 %; +++, >75 %.

† Estimated amount of protein present relative to WT. ?, Results that cannot be clearly interpreted owing to lack of mAb A4 binding.

‡ Fusion activity as a percentage of WT. −, Values <5 % of WT are indicated as being negative.

§ Infectivity as a percentage of WT. −, Values <2 % of WT are indicated as being negative.

To check for stable expression of mutated glycoproteins, lysates of HEK-293T cells transiently transfected with WT and mutant E1E2 were tested by *Galanthus nivalis* agglutinin (GNA) ELISA for reactivity to anti-E1 and anti-E2 mAbs that recognize linear epitopes. Upon serial dilution of lysates we saw a reasonably constant relationship between the dilution and the signal, showing that the assay was not saturated and that the signal was dependent on the concentration of glycoprotein present in the lysate (not shown). All mutants gave a signal that was at least 50 % of the WT signal observed with E1 mAbs H-111 and/or AP21.010. Similarly, all mutants (except for the negative control mutant *in*515-STOP) gave a signal that was at least 90 % that of WT with mAb AP33, and at least 65 % that of WT with mAb ALP98 (Table 2). Given that all the transiently expressed mutated glycoproteins were of similar concentration and stability in crude cell lysates, the relative effect of the mutations on structure and function could be analysed in the assays described below.

### Antigenic analysis of mutated E1E2 reveals a strong folding determinant in the E2 glycoprotein

To analyse the influence of insertions on protein folding, lysates of HEK-293T cells transiently transfected with WT and mutant E1E2 were tested by GNA ELISA for reactivity with conformation-sensitive murine anti-E2 mAbs H35, H48 and H53, and human anti-E2 mAbs CBH-4B, CBH-4D, CBH-4G, CBH-5 and CBH-7. The concentration of each lysate was normalized on the basis of reactivity with mAbs AP33 and ALP98. Several mutants were clearly much less reactive with the conformation-sensitive mAbs than WT. To stringently define the mutations that had the greatest effect, the data were analysed statistically and those where *P*<0.01 were considered to be significant.

**Fig. 2.**
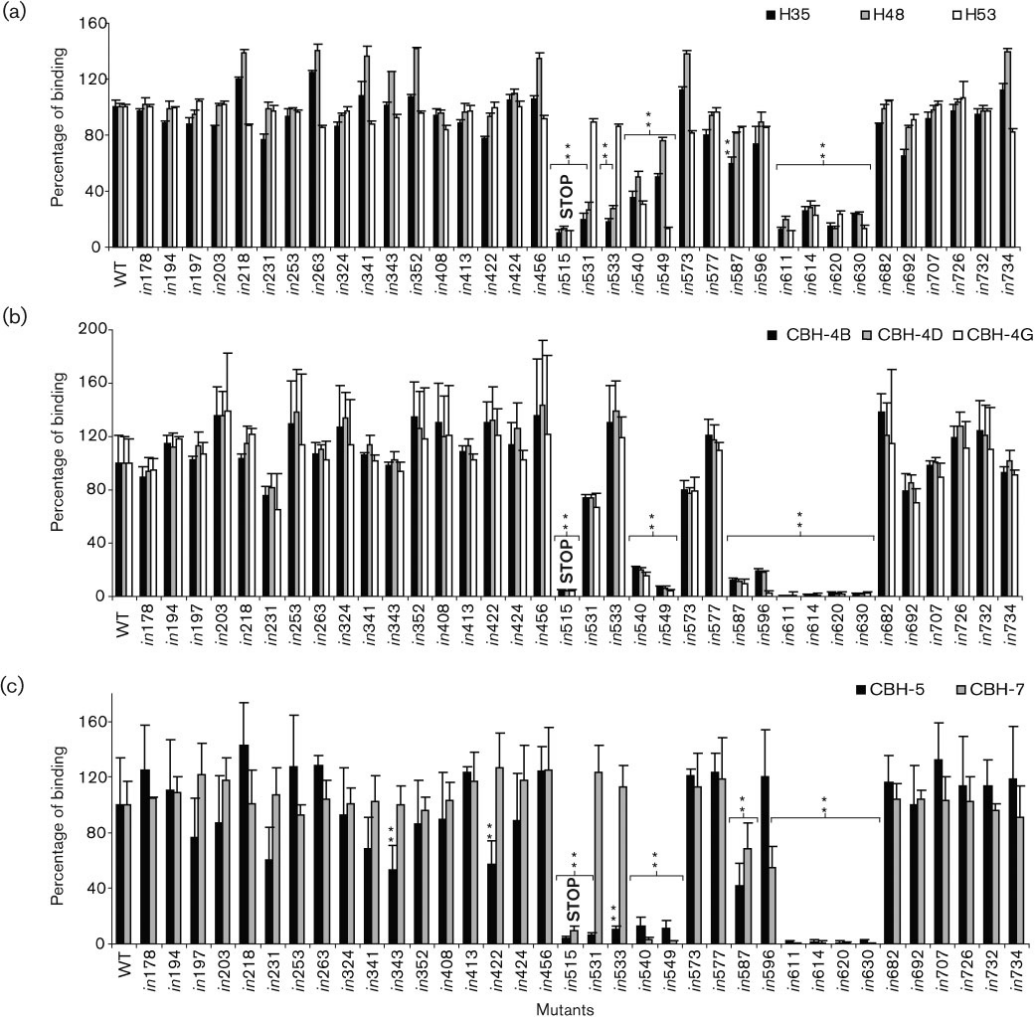
Folding analysis of mutated E1E2 using conformation-sensitive mAbs. WT and mutated E1E2 glycoproteins (numbered as in Fig. 1) in lysates of transfected HEK-293T cells, normalized for reactivity with mAbs AP33 and ALP98, were captured by GNA and probed with (a) mouse anti-E2 mAbs H35, H48 and H53; (b) human anti-E2 mAbs CBH-4B, -4D and -4G; and (c) CBH-5 and CBH-7. The reactivity of each mutant is presented as a percentage of the signal for WT, after subtracting the signal obtained with mock-transfected cell lysate. Values shown are the means and sd of three independent assays. **, Values that differ significantly from WT (*P*<0.01).

**Fig. 6.**
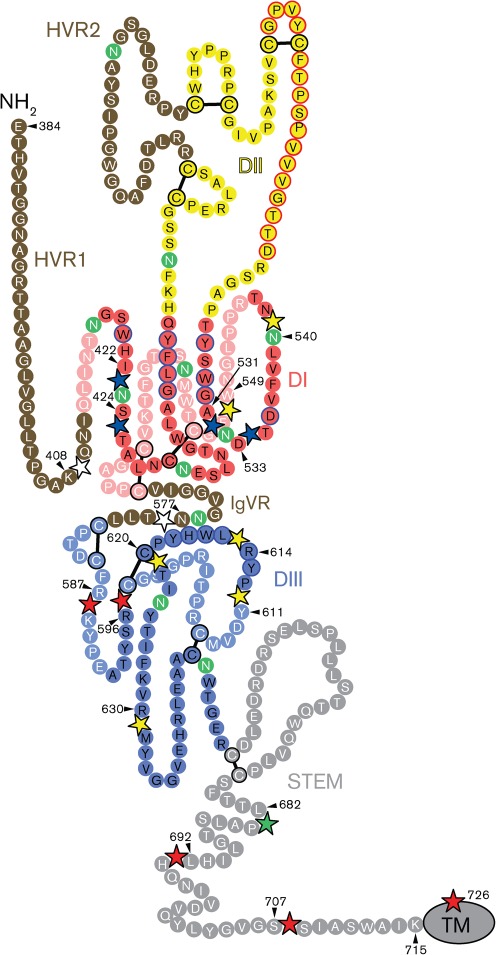
Localization of insertions on the model of HCV E2. The linear sequence of H77c E2 is represented as a chain of beads threaded onto a class II fusion protein fold. Structural domains are coloured red (DI), yellow (DII) and blue (DIII), the stem region is grey and the unstructured regions (HVR1, HVR2 and IgVR) are brown. Black font on bright colour indicates foreground residues and white font on pale colour indicates background residues. The transmembrane domain (TM) is represented by an ellipse at the C terminus. Glycosylation sites are coloured green. CD81-binding residues are circled in blue. Putative fusion-loop residues are circled in red. Disulphide bonds are shown as black bars. Intercalated stars show the position of insertions that retain infectivity (white), and those that affect protein folding (yellow), heterodimer formation (red), CD81 binding (blue) and fusion (green). The amino acid residue immediately N-terminal to each insertion is numbered. Adapted from Krey *et al.* (2010).

The most striking result was that four consecutive mutants in the region between amino acid residues 611 and 631 were not recognized by any of the conformation-sensitive mAbs (Fig. 2 and Table 2), which shows that these mutants were misfolded. This region is highly conserved in different HCV genotypes and has been implicated in dimerization (Yagnik *et al.*, 2000), CD81 binding (Roccasecca *et al.*, 2003; Rothwangl *et al.*, 2008), membrane fusion (Lavillette *et al.*, 2007), binding of conformation-sensitive mAbs and infectivity (Iacob *et al.*, 2008; Rothwangl *et al.*, 2008). A new model of the tertiary structure of E2 assigns amino acid residues 581–651 to a distinct structural domain (DIII) stabilized by two long-range disulphide bonds, C597–C620 and C607–C644 (Krey *et al.*, 2010, reproduced in Fig. 6). We propose that the region bounded by these disulphide bonds comprises a critical folding determinant whose structure is essential for the correct folding and functioning of E2.

Likewise, there was a highly significant reduction in binding (*P*<0.01) of all eight mAbs with mutants *in*540 and *in*549 (Fig. 2 and Table 2), showing that this region, which forms part of the central DI domain in the model (Fig. 6), is also essential for correct protein folding. It has been observed that mutation of the glycosylation site at N540 consistently reduces the affinity of various neutralizing antibodies, including CBH-5, CBH-7 and H48 (Helle *et al.*, 2007; Owsianka *et al.*, 2008), suggesting that absence of the glycan induces a local change in conformation. It is not surprising, therefore, that an insertion at this position has a similar effect.

In contrast, mutations in two other regions, amino acid residues 531–534 and 587–597, had a more selective effect. Insertions *in*531 and *in*533 severely reduced the binding of H35, H48 and CBH-5, but had little or no effect on the binding of the other five mAbs. Insertion *in*587 severely reduced the binding of CBH-4B, -4D and -4G and moderately that of H35, CBH-5 and CBH-7, but had no effect on H48 and H53. Similarly, *in*596 severely reduced the binding of CBH-4B, -4D and -4G, and moderately that of CBH-7, but had no effect on the other four mAbs (Fig. 2 and Table 2). Therefore, these insertions do not cause E2 to misfold, but probably disrupt antibody epitopes or else induce a very local conformational change that affects the binding of some mAbs but not others. The mAbs used in this study have been fairly well characterized, but our knowledge of the residues that contribute to their conformational epitopes is incomplete. The five human mAbs map to three distinct domains: CBH-4B, -4D and -4G to domain A, CBH-5 to domain B and CBH-7 to domain C (Hadlock *et al.*, 2000; Keck *et al.*, 2004a). Domain A mAbs are non-neutralizing, whereas domain B and C mAbs inhibit E2–CD81 binding and neutralize infectivity (Keck *et al.*, 2004a; Owsianka *et al.*, 2008). Mouse mAbs H35 and H48 are neutralizing, while H53 is non-neutralizing (Bartosch *et al.*, 2003a; Cocquerel *et al.*, 1998; Dubuisson *et al.*, 1994). The neutralizing mAbs H35, H48 and CBH-5 have a similar footprint, and Gly-530 is a critical binding residue for all three antibodies (Keck *et al.*, 2008; Owsianka *et al.*, 2006, 2008). This suggests that insertions *in*531 and *in*533 directly disrupt an important component of their common epitope, with little impact on E2 conformation. On the other hand, insertions *in*587 and *in*596 affect the binding of mAbs that recognize different epitopes, so they may induce a local conformational change that does not alter the overall folding of E2.

### Influence of insertions on E1E2 heterodimer formation

**Fig. 3.**
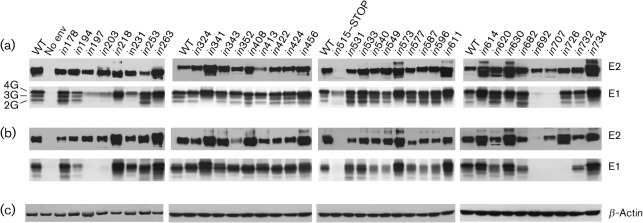
Heterodimerization of mutated E1E2. WT and mutated E1E2 glycoproteins (numbered as in Fig. 1) in lysates of transfected HEK-293T cells were immunoprecipitated with anti-E2 serum R646, resolved by (a) reducing and (b) non-reducing SDS-PAGE, and analysed by immunoblotting with anti-E2 mAbs (AP33 and ALP98) and anti-E1 mAb A4. Bands corresponding to different glycoforms of E1 (E1-2G, -3G and -4G) are indicated. No env, Mock-transfected control cells. (c) β-Actin immunodetection to check the protein concentration of cell lysates.

We investigated the ability of mutated E1E2 that was transiently expressed in HEK-293T cells to assemble into non-covalent heterodimers. The proteins were immunoprecipitated with rabbit polyclonal anti-E2 serum R646, separated by using SDS-PAGE under reducing and non-reducing conditions, and detected by immunoblotting. The WT heterodimer consisted of E2, which migrated predominantly as a single band, associated non-covalently with different glycoforms of E1 (Fig. 3). Most of the mutants were co-precipitated by anti-E2 serum R646, although some variation in migration and glycosylation patterns was observed.

Analysis on non-reducing gels showed that E2 mutants *in*540, *in*549, *in*587, *in*596, *in*707 and *in*726 yielded lower levels of co-precipitated E1, which indicated impaired E1E2 heterodimer formation (Fig. 3b and Table 2). In E2 mutant *in*692 (and also in the truncated *in*515-STOP mutant), we observed reduced immunoprecipitation of E2 and no detectable co-precipitation of E1. The apparent lack of E1 in the case of mutants *in*197 and *in*203 is caused by these insertions disrupting the epitope (aa 197–207) of mAb A4, which was used for detection, and should not be interpreted as a lack of heterodimerization. The only E1 mutant that showed a reduced level of heterodimerization is *in*194, but, because the insertion is very close to the A4 epitope, this result cannot be interpreted with certainty.

The reduced heterodimerization observed for mutants *in*540 and *in*549 is probably a consequence of these mutants being misfolded, as discussed above. Mutants *in*587, *in*596, *in*692, *in*707 and *in*726 are correctly folded, although *in*587 and *in*596 may induce a local conformational change that does not impact on the overall folding of E2. These mutants therefore indicate two regions of E2 that are involved in heterodimerization: amino acids 587–597 and 692–727. The latter region spans the membrane-proximal heptad repeat region and transmembrane domain of E2. This agrees with previous work, which shows that formation of a non-covalent heterodimer depends on interaction between the transmembrane domains of both E1 (aa 352–383) and E2 (aa 718–746) (reviewed by Lavie *et al.*, 2007), and that residues in the conserved hydrophobic membrane-proximal heptad repeat region of E2 (aa 675–699) are also required (Drummer & Poumbourios, 2004). The region represented by mutants *in*587 and *in*596 is located in domain DIII of the model of E2 (Krey *et al.*, 2010, Fig. 6), and could form part of the interface with E1 or contribute to stabilizing the complex. In summary, our results confirm the role of the transmembrane domain and the membrane-proximal hydrophobic region of E2 in heterodimer formation, and also identify another region of E2 (aa 587–597) that may be involved in complex assembly.

### CD81 binding properties of mutated E1E2

**Fig. 4.**
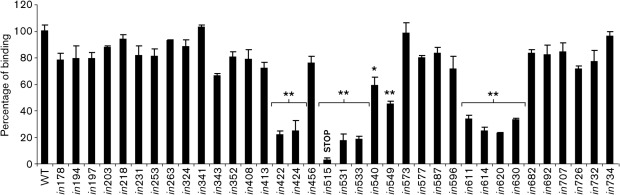
CD81 binding of mutated E1E2. Immobilized glutathione *S*-transferase (GST)–CD81 large extracellular loop (LEL) was used to capture WT and mutated E1E2 glycoproteins (numbered as in Fig. 1) from lysates of transfected HEK-293T cells. The concentration of lysate was normalized for reactivity with AP33 and ALP98 mAbs in a GNA ELISA. CD81-bound E2 was detected by using anti-E2 serum R646. Binding of each mutant is presented as a percentage of the signal for WT, after subtracting the signal obtained with mock-transfected cell lysate. Values shown are the means and sd of assays performed in triplicate. Consistent data were obtained in two or more independent experiments. Asterisks indicate values that differ significantly from WT: **, *P*<0.01; *, *P* = 0.04.

The mutants were tested for their ability to bind CD81, an essential receptor for virus entry into target cells (Pileri *et al.*, 1998). All mutants with insertions in E1 bound CD81 with approximately WT efficiency, whereas a significant decrease in CD81 binding was observed with several E2 mutants (Fig. 4 and Table 2). A 40–55 % reduction in binding was seen with mutants *in*540 and *in*549, while all mutants in the region between amino acid residues 611 and 631 resulted in a 70–80 % reduction of CD81 binding. This is almost certainly a consequence of protein misfolding caused by these insertions, as described above, rather than a direct effect. The greatest reduction (75–85 %) in CD81 binding was observed for mutants *in*422, *in*424, *in*531 and *in*533. These mutants are correctly folded, since they are recognized by most of the conformation-sensitive mAbs (Fig. 2 and Table 2), and therefore we conclude that these four insertions directly affect the CD81-binding site.

Several non-contiguous amino acid residues of E2 have been identified as being important for CD81 receptor binding: Trp-420 and amino acid residues 436–443, 527–535 and 613–618 (Drummer *et al.*, 2006; Owsianka *et al.*, 2006; Roccasecca *et al.*, 2003; Rothwangl *et al.*, 2008), indicating that the binding site is conformation-sensitive and is dependent upon correct E2 folding. Our study shows that mutations *in*531 and *in*533 disrupt the binding both of soluble CD81 and of neutralizing antibodies H35, H48 and CBH-5 (Table 2). These mutations are likely to interfere directly with the accessibility of Trp-529, Gly-530 and Asp-535, which are critical CD81-binding residues. Similarly, the CD81-binding defect of mutants *in*422 and *in*424 might result from the proximity of these inserts to Trp-420. These four mutations are all located in close proximity to each other on domain DI (Fig. 6) (Krey *et al.*, 2010), highlighting the importance of this domain for receptor binding and virus neutralization.

### Insertions in E1 and E2 have a major influence on HCV pseudo-particle infectivity

**Fig. 5.**
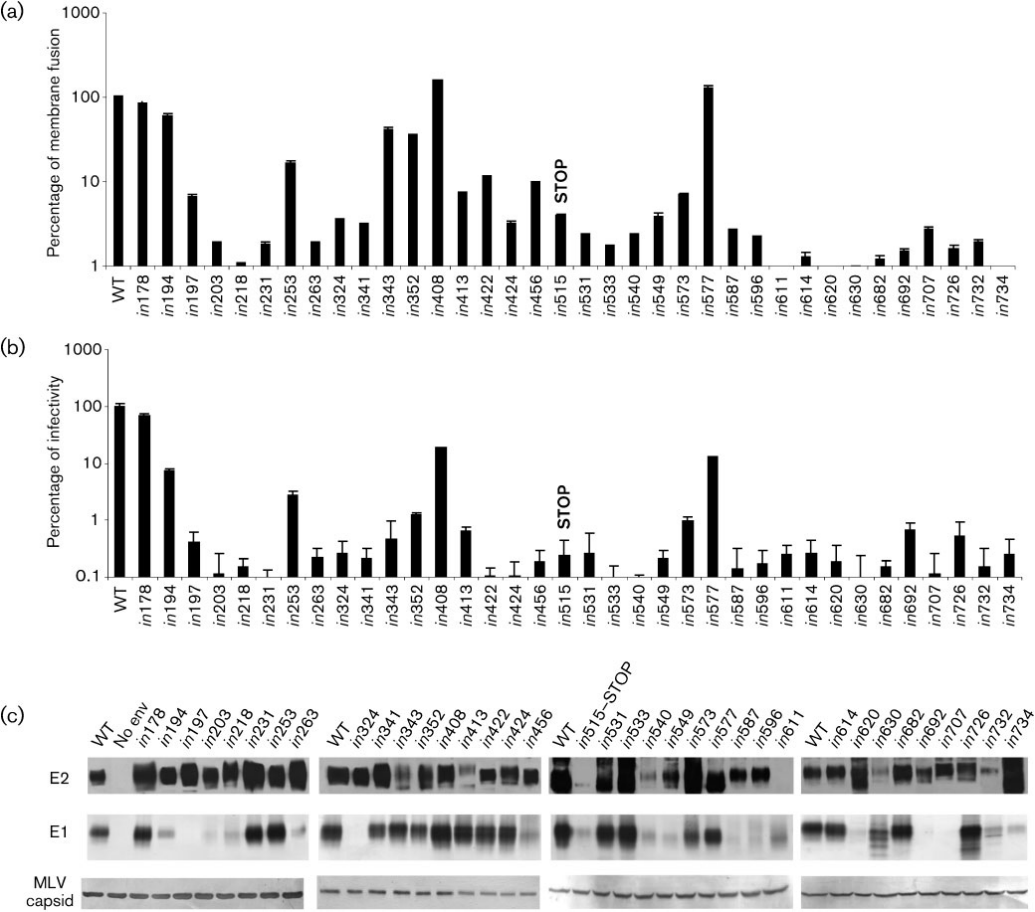
Membrane fusion activity and HCVpp infectivity of mutated E1E2. (a) HEK-293T cells expressing WT or mutated E1E2 glycoproteins (numbered as in Fig. 1), together with luciferase under the control of the inducible HIV-1 LTR promoter, were co-cultivated with Huh7-J6 indicator cells expressing HIV-1 Tat protein. Fusion was induced by low-pH treatment and 24 h later luminescence was measured. The value for each mutant is expressed as a percentage of the signal for WT, after subtracting the signal obtained with donor cells devoid of HCV glycoproteins. Values shown are the means and sd of assays performed in triplicate. Consistent data were obtained in two or more independent experiments. (b, c) Pseudoparticles were generated that incorporated WT and mutated E1E2 glycoproteins (numbered as in Fig. 1), or no envelope glycoproteins (No env). (b) The pseudoparticles were used to infect Huh-7 cells, and infectivity was measured after 4 days by using a GFP reporter assay. The infectivity of each mutant is expressed as a percentage of the signal for WT, after subtracting the infectivity of the no-envelope control. Values shown are the means and sd of assays performed in triplicate. Similar results were obtained by using a luciferase reporter. (c) Concentrated pseudoparticles were immunoprecipitated with polyclonal anti-E2 serum R646, resolved by non-reducing SDS-PAGE and analysed by immunoblotting for E2 (mAbs AP33 and ALP98), E1 (mAb A4) and MLV capsid (CRL-1912).

Having established that a number of insertions had multiple effects on both the structure and function of E1E2, we related these effects to infectivity by generating recombinant HCV pseudo-particles (HCVpp) bearing WT or mutated glycoproteins and testing them for infectivity of Huh-7 cells. Most of the insertions in both E1 and E2 resulted in a dramatic decrease or a complete loss of infectivity (Fig. 5b and Table 2). Only four mutants maintained clearly demonstrable levels of cell entry, although all were significantly lower than WT: *in*194 (7 %), *in*253 (3 %), *in*408 (20 %) and *in*577 (13 %). Only the control mutant *in*178, with an insertion outside the glycoprotein sequence, maintained normal infectivity (70 % of WT), in keeping with its WT characteristics in all other assays.

To assess whether the lack of infectivity could be explained by impaired incorporation of glycoproteins, sucrose-cushion-concentrated HCVpp were immunoprecipitated with anti-E2 serum R646 and analysed by using non-reducing SDS-PAGE followed by immunoblotting for E1, E2 and murine leukemia virus (MLV) Gag protein (Fig. 5c). Significant differences in E1E2 incorporation levels were found in approximately half of the mutant HCVpp. Specifically, very low levels of E1 incorporation were seen in mutants *in*218, *in*263 and *in*324, and very low levels of one or both glycoproteins in mutants *in*456, *in*540, *in*549, *in*587, *in*596, *in*611, *in*620, *in*630, *in*692, *in*707, *in*732, *in*734 and the truncated control *in*515-STOP. As previously, the apparent lack of E1 in the case of mutants *in*194, *in*197 and *in*203 is caused by these insertions disrupting the epitope of mAb A4, and should not be interpreted as a lack of incorporation. All of the misfolded mutants produced defective HCVpp except for *in*614, which, intriguingly, incorporated the non-covalent heterodimer at WT levels.

### Fusion competence of mutated E1E2

We reasoned that non-infectious mutants that incorporated normal levels of glycoproteins might have a reduced membrane fusion capability. To perform a cell-based fusion assay, HEK-293T donor cells were co-transfected with plasmids encoding E1 and E2 glycoproteins, together with a luciferase reporter gene under the control of the human immunodeficiency virus (HIV)-1 LTR. Donor cells were co-cultivated with Huh7-J6 indicator cells expressing HIV-1 transcription transactivator protein (Tat), and fusion was induced by lowering the pH to 5. Under these conditions donor cells bearing WT E1E2 gave a robust signal of 10^5^ relative luminescence units (RLU), while donor cells lacking HCV glycoproteins gave a signal of <10^3^ RLU. To further validate the assay, donor cells expressing WT E1E2 and luciferase reporter were combined with indicator cells devoid of Tat transactivator, and this combination also resulted in a signal of <10^3^ RLU.

We found a clear correlation between the infectivity profile of the mutants and their ability to mediate cell fusion (Fig. 5a, b). Most of the mutants were severely impaired or unable to mediate fusion, but several retained a significant proportion of WT fusogenic activity, specifically E1 mutants *in*194 (61 %), *in*253 (17 %), *in*343 (42 %) and *in*352 (35 %), and E2 mutants *in*408 (159 %) and *in*577 (130 %). Only those mutants (*in*194, *in*408 and *in*577) that had levels of fusogenic activity close to or greater than WT retained moderate (7 % to 20 %) HCVpp infectivity (Table 2). This may be because both assays depend on the expression of functional glycoproteins on the plasma membrane of HEK cells. All three infectious mutants have inserts in highly variable regions: *in*194 is at the very N terminus of E1, *in*408 is in the first hypervariable region (HVR1) of E2 and *in*577 is in the intergenotypic variable region (igVR) of E2. HVR1 is dispensable for HCV infection but viruses lacking this region or carrying an insertion in it have been shown to be less infectious (Bankwitz *et al.*, 2010; Forns *et al.*, 2000; Wakita *et al.*, 2005). The igVR is extremely variable in sequence and also in length (10–15 aa, depending on genotype), but all isolates contain a single conserved *N*-linked glycosylation site at approximately amino acid position 576 (Humphreys *et al.*, 2009; McCaffrey *et al.*, 2007). Interestingly, the igVR is predicted to function as an inter-domain hinge region, which allows the molecule to flex and go through the large conformational change that is necessary to mediate fusion (Krey *et al.*, 2010). Our data suggest that this region is so plastic that it can tolerate an increase in length from 11 to 16 aa and also the loss of an *N*-linked glycan (the glycosylation site is disrupted in the *in*577 mutant). This is in keeping with previous work, which showed that HCVpp lacking this glycosylation site retained infectivity (Goffard *et al.*, 2005; Helle *et al.*, 2007).

Fusion occurs upon endocytosis in early endosomes and requires an acidic pH, which induces a conformational rearrangement of the envelope proteins (reviewed by Helle & Dubuisson, 2008). Mutagenesis of conserved residues in putative fusion-active regions has identified three regions that may be functionally important: amino acid residues 259–286 in E1, and 416–430 and 600–620 in E2 (Drummer *et al.*, 2007; Lavillette *et al.*, 2007; Russell *et al.*, 2009), and the latest structural model predicts that the fusion peptide comprises amino acid residues 502–520 in E2 (Krey *et al.*, 2010). Several of our mutants (*in*263, *in*422, *in*424, *in*611, *in*614 and *in*620) have insertions that disrupt one of these regions, and they are all negative for membrane fusion. However, each of them is deficient in at least one other function: mutant *in*263 does not incorporate normal levels of E1 into HCVpp, and is therefore unlikely to display normal levels of E1 on the plasma membrane of transfected HEK cells; mutants *in*422 and *in*424 do not bind CD81; while mutants *in*611, *in*614 and *in*620 are misfolded. In each case the lack of fusion is very likely to be an indirect effect.

Similarly, the lack of fusogenic activity in most of the other mutants can be ascribed to a defect in another function. However, mutants *in*231, *in*341 and *in*682 have WT characteristics in every assay other than fusion and infectivity, and therefore these insertions may be having a direct effect on the mechanism of membrane fusion. Mutant *in*682 is particularly interesting because this insertion disrupts the conserved hydrophobic heptad-repeat region in the flexible, membrane-proximal ‘stem’ of E2 (Fig. 6). By analogy with the well-characterized class I fusion proteins of influenza and HIV-1, the stem region of flavivirus class II fusion proteins is proposed to play a key role in membrane fusion by interacting closely with a region of the protein close to the fusion loop and thus stabilizing the post-fusion ‘hairpin’ conformation (reviewed by Harrison, 2008; Kielian & Rey, 2006). Drummer & Poumbourios (2004) demonstrated that conserved residues in the hydrophobic heptad repeat were essential for HCV cell entry, and proposed that this region of E2 is functionally homologous to the stem of flavivirus E glycoproteins. The characteristics of mutant *in*682 provide direct evidence that this region plays a role in membrane fusion.

A tabulated summary of all the data obtained in this analysis (Table 2) shows the relationships between the fundamental properties of correct folding and heterodimerization and the higher-level functions of receptor binding, HCVpp incorporation, fusion and infectivity. Fig. 6 maps the insertions that affect specific functions on a model of the E2 protein (Krey *et al.* 2010). Unfortunately, the mutagenesis was relatively less informative about E1 in that none of the E1 insertions that we obtained affected heterodimer formation. However the insert at amino acid position 324, which disrupts a very conserved region, dramatically reduced incorporation of E1 into HCVpp. It is striking that membrane fusion and HCVpp infectivity were severely affected by all insertions in E1, except for one at the very N terminus, thus emphasizing the involvement of E1 in the fusion and entry process. The effects of insertions within E2 point to the following structure–function relationships: (i) correct folding of E2 requires the structural integrity of regions 611–631 and 540–549; (ii) E1E2 heterodimerization involves regions 587–597 and 692–727; (iii) CD81 binding is disrupted by insertions at amino acid residues 422–425 and 531–534; (iv) incorporation of E1E2 into HCVpp is reduced by insertions at residues 456 and 732–735, which also abrogate membrane fusion; and (v) insertion at Leu-682 specifically disrupts fusion. Overall, our study shows that insertions at most sites in the E1E2 glycoprotein complex abrogate infection. A similar observation was made in the context of whole-genome analysis, which showed that the E1E2 sequence is considerably less tolerant of insertions than most other regions of the HCV genome (Arumugaswami *et al.*, 2008). Nevertheless, we have identified positions in the E1 (aa 194) and E2 glycoproteins (aa 408 and 577, Fig. 6) that can be modified by insertion of a short peptide without loss of function. A small affinity tag inserted at these positions could be used for purification of native E1E2 heterodimer or even functional analysis of HCV virions.

## Methods

### 

#### Plasmid constructs.

Plasmids encoding HCV genotype 1a strain H77c E1E2 (aa 137–746 of the viral polyprotein; Yanagi *et al.*, 1997), the MLV Gag–Pol, and the MLV transfer vectors encoding GFP or luciferase reporter have been described previously (Bartosch *et al.*, 2003a; Owsianka *et al.*, 2005). The MLV transfer vector pCNC-TAT (Ikeda *et al.*, 2003) and the plasmid pLTR-luc were a kind gift from Yasuhiro Takeuchi (University College London, UK) and Dan Littman (New York University School of Medicine, USA), respectively.

#### Antibodies.

Anti-E2 mAbs AP33, ALP98, H35, H48 and H53, anti-E2 rabbit polyclonal serum R646, and anti-E1 mAbs A4 and AP21.010 have been described previously (Clayton *et al.*, 2002; Cocquerel *et al.*, 1998, 2003; Dubuisson *et al.*, 1994; Owsianka *et al.*, 2001). Human mAbs H-111 (anti-E1), and CBH-4B, CBH-4D, CBH-4G, CBH-5 and CBH-7 (all anti-E2) have been reported previously (Hadlock *et al.*, 2000; Keck *et al.*, 2004b). MLV capsid-specific mAb was obtained from rat hybridoma cells (ATCC accession no. CRL-1912). Anti-β-actin mAb was purchased from Santa Cruz Biotechnology.

#### Construction of mutants.

Random mutations in HCV E1E2 glycoproteins were generated by linker-scanning mutagenesis using a Tn-7-based transposon mutagenesis kit (GPS-LS Linker Scanning System; NEB). In brief, the plasmid encoding HCV genotype 1a strain H77 E1E2 cDNA was used as a target template in an *in vitro* transposition reaction with a donor plasmid containing a kanamycin selection marker flanked by two attachment sites (*Tn7L* and *Tn7R*) for transposase. After transformation into *Escherichia coli* strain DH5-α, plasmids from kanamycin-resistant colonies were screened by restriction digestion to exclude insertions in the vector or promoter DNA. Selected plasmids with insertions in the E1 and E2 gene sequences were digested with *Pme*I, to remove most of the transposon containing the selection marker, and self-ligated, resulting in 15-base-pair insertions. The nucleotide sequence of the insertions was TGTTTAAACA(N)_5_, where (N)_5_ are the duplicated nucleic acid residues of the target sequence at the insertion site. All the mutants were sequenced to identify the position and sequence of the insertions.

#### Cell lines.

Human hepatoma Huh-7 cells and human epithelial kidney HEK-293T cells were propagated as described previously by Owsianka *et al.*, (2005). Huh7-J6 indicator cells were generated by transducing Huh-7 cells with HIV-1 Tat expression vector pCNC-TAT and selecting in medium containing 500 µg G418 ml^−1^.

#### HCVpp infectivity and glycoprotein incorporation.

HCVpp were produced as described previously (Bartosch *et al.*, 2003a). In brief, HEK-293T cells were co-transfected with plasmids encoding WT or mutated HCV E1E2 cDNA, the MLV Gag–Pol packaging vector and the MLV–GFP or MLV–luc transfer vector. Control pseudoparticles were generated by omitting the glycoprotein construct. At 2 days post-transfection, the pseudoparticle-containing medium was harvested, clarified, filtered (0.45 µm pore size) and used to infect Huh-7 cells. After 4 h incubation at 37 °C, the inoculum was replaced with fresh medium, the cells incubated for 4 days and then the cells were analysed for reporter-gene expression by flow cytometry on a FACSCalibur (GFP) or by using Bright-Glo (Promega) and a Chameleon II plate reader (luciferase).

To analyse incorporation of mutated E1E2, HCVpps were concentrated and purified by ultracentrifugation over a 20 % sucrose cushion (116 000 ***g***, 3 h, 4 °C). The pellets were resuspended in PBS (pH 7.4) to concentrate viral particles 20-fold and incubated with anti-E2 R646 rabbit antiserum for 2 h at 4 °C. Antibody complexes were precipitated by using protein A–Sepharose (Sigma-Aldrich) and analysed by SDS-PAGE (10 % acrylamide gels) followed by immunoblotting with E1 (A4), E2 (AP33 and ALP98) and MLV-gag mAbs, followed by HRP-conjugated anti-species secondary antibodies and enhanced chemiluminescence detection (SuperSignal West Dura Chemiluminescent Substrate; Thermo Scientific).

#### Analysis of heterodimer formation.

HEK-293T cells were transiently transfected with plasmids encoding WT or mutated HCV E1E2 cDNA. Mock-transfected cells served as a negative control. At 48 h post-transfection, cells were incubated for 30 min with lysis buffer (20 mM Tris/HCl, pH 7.4, 150 mM NaCl, 1 mM EDTA, 1 % Triton X-100, 20 mM iodoacetamide). Lysates were clarified by centrifugation and incubated with anti-E2 R646 rabbit antiserum for 2 h at 4 °C. The antibody complexes were precipitated by using protein A–Sepharose and analysed by SDS-PAGE and immunoblotting with E1 and E2 mAbs, as described above.

#### GNA ELISA.

Detection of E1E2 by ELISA was performed as described by Patel *et al.* (2000). Briefly, Immulon II plates coated with GNA were used to capture glycoproteins from lysates of HEK-293T cells transiently expressing WT or mutated E1E2, prepared as described above. Lysate from mock-transfected cells served as a negative control. Bound proteins were detected by using appropriate antibodies followed by anti-species IgG–HRP conjugate and TMB substrate (Sigma). Statistical analysis was carried out using a *Z*-test to assess whether there was a significant difference between the reactivity of each individual mutant and the mean of the whole dataset, the null hypothesis being that all mutants were identical to WT.

#### CD81 binding assay.

Microtitre plates (Costar 3590) coated with 0.5 µg human CD81–LEL–GST fusion protein ml^−1^ were used to capture glycoproteins from lysates of HEK-293T cells transiently expressing WT or mutated E1E2, prepared as described above. Lysate concentrations were normalized for E2 reactivity with mAbs AP33 and ALP98 in GNA ELISAs. Lysate from mock-transfected cells served as a negative control. The CD81-bound glycoproteins were detected by using anti-E2 serum R646 and anti-rabbit IgG–HRP conjugate. Statistical analysis was carried out as above.

#### HCV cell–cell fusion.

The assay was performed essentially as previously described (Kobayashi *et al.*, 2006; Lavillette *et al.*, 2007). Briefly, HEK-293T cells were co-transfected with plasmids encoding WT or mutated E1 and E2 glycoprotein sequences and HIV-2 LTR luciferase reporter gene (pLTR-luc). Control cells were transfected with (i) WT E1E2 but no reporter and (ii) empty phCMV vector and pLTR-luc. After 12 h incubation at 37 °C, transfected cells were reseeded at 10^5^ cells per well in six-well plates, together with Huh7-J6 indicator cells or Huh-7 control cells at 4×10^5^ cells per well. After 24 h of co-cultivation, the cells were washed with PBS, incubated for 5 min in a pH 5.0 fusion buffer (130 mM NaCl, 15 mM sodium citrate, 10 mM MES, 5 mM HEPES) and washed three times with medium. Luciferase activity was measured 24 h later by using Bright-Glo (Promega).
